# What was visualized? A method for describing content of performance summary displays in feedback interventions

**DOI:** 10.1186/s12874-020-00951-x

**Published:** 2020-04-23

**Authors:** Dahee Lee, Veena Panicker, Colin Gross, Jessica Zhang, Zach Landis-Lewis

**Affiliations:** Department of Learning Health Sciences, University of Michigan Medical School, 1161J NIB, 300 N., Ingalls Street, SPC 5403, Ann Arbor, MI 48109-5403 USA

**Keywords:** Audit and feedback, Visualization, Content analysis, Clinical quality improvement

## Abstract

**Background:**

Visual displays such as charts and tables may significantly moderate the effects of audit and feedback interventions, but the systematic study of these intervention components will likely remain limited without a method for isolating the information content of a visual display from its form elements. The objective of this study is to introduce such a method based on an application of visualization frameworks to enable a systematic approach to answer the question, “What was visualized?” in studies of audit and feedback.

**Methods:**

The proposed method uses 3 steps to systematically identify and describe the content of visual displays in feedback interventions: 1) *identify displays*, 2) *classify content*, and 3) *identify elements*. The use of a visualization framework led us to identify information content types as representations of *measures* (metrics or indicators), *ascribees* (feedback recipients and comparators), *performance levels*, and *time intervals*. We illustrate the proposed method in a series of 3 content analyses, one for each step, to identify visual displays and their information content in published example performance summaries.

**Results:**

We analyzed a convenience sample of 44 published studies of audit and feedback. Through each step, two coders had good agreement. We identified 42 visual displays of performance, containing 6 unique combinations of content types. What was visualized most commonly in the sample was performance levels across a recipient and comparators (i.e. ascribees) for a single measure and single time interval (*n* = 16). Content types varied in their inclusion of measures, ascribees, and time intervals.

**Conclusions:**

The proposed method appears to be feasible to use as a systematic approach to describing visual displays of performance. The key implication of the method is that it offers more granular and consistent description for empirical, theoretical, and design studies about the information content of feedback interventions.

## Background

Audit and feedback (A&F) is a widely-used implementation strategy that has attracted decades of research attention in more than 150 trials [1]. A&F trials generally yield moderate (4% absolute) improvements in desired practice, but mixed effects demonstrate potential for large positive effects under ideal conditions [2]. To understand how to achieve larger effects, researchers have sought to identify mechanisms through which A&F influences clinical practice [1].

Evidence shows that the visual display of performance in feedback interventions can significantly moderate its effects on clinical practice [3, 4]. Therefore, important mechanisms of action for A&F may be related to the use of visualizations [5–7]. We understand visualizations to include charts, tables, and hybrid displays of graphical elements in tables [8], all of which are commonly used in feedback reports and clinical quality dashboards [9, 10]. The effectiveness of these visualizations can depend on various factors [11, 12], including visual characteristics like the type of chart used [13], and characteristics of the people who receive feedback, such as numeracy and graph literacy [14, 15].

However, a fundamental barrier to studying these factors is the lack of systematic description of the information content, in other words *what is being visualized*, in a performance summary. To our knowledge, no systematic method for describing the content of visualized clinical performance information has been developed. However, a framework called Relational Information Displays [8] enables a unified and systematic approach to the description of charts and tables, and is the only such framework to our knowledge that is centered around relationships between format and content elements.

The Relational Information Displays framework enables two kinds of description of charts and tables: 1) description of the *visual elements* of a display (e.g. points, lines, areas, shapes, colors, positions and orientation) and 2) description of the representations that a feedback recipient attributes to the visual elements, which we refer to as *content elements*. For example, in a bar chart, two bars may appear (two visual elements) that have two different content elements: One bar represents *your hospital* and another bar represents *an average for hospitals in your region*. By carefully isolating the characteristics of visual and content elements, the Relational Information Displays framework also enables description of the relationships between visual and content elements, which can be used to optimize displays for cognitive processing via the human visual system, depending on shared qualities of visual and content elements [8].

The objective of this study is to propose a method for identifying the content of visual displays of clinical performance, to enable a systematic approach to answering the question “What was visualized?”, to isolate important aspects of visualizations in A&F research. We illustrate the use of this method using content analysis of example feedback reports from published studies of A&F.

## Methods

We propose a method of describing visual displays and their content in performance summaries. We use the term *performance summary* to refer to a kind of communication about performance data, typically in the form of a document or static page of a web site (i.e. a feedback report), and that can exist in digital or paper form. We consider clinical quality dashboards to also contain performance summaries that could appear static form, therefore we do not distinguish between summaries of performance in feedback reports and dashboards.

**Fig. 1:**
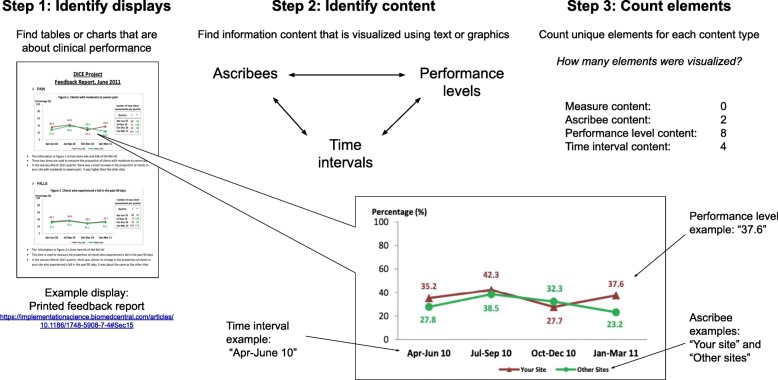
Describing visualized content of a performance summary display in a printed feedback report

**Fig. 2:**
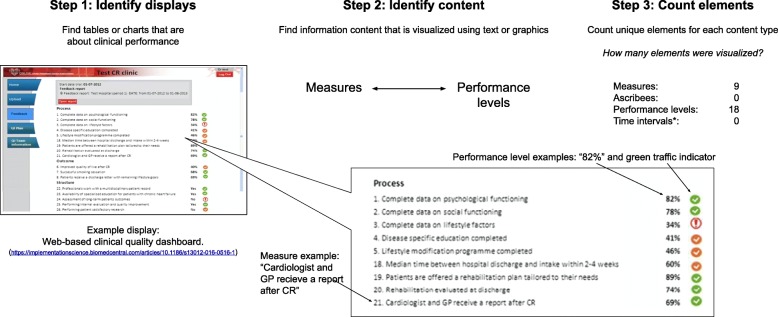
Describing visualized content of a performance summary display in a clinical quality dashboard

The proposed method includes 3 steps: 1) *identify displays*, 2) *classify content*, and 3) *identify elements*. We selected example feedback reports to contrast content types in Figs. 1 and 2, which contain different chart types (line chart vs hybrid table with graphical elements), delivery format (printed report vs web-based dashboard), and content types. We describe coding for each step using directed content analysis, which can be validated using the agreement of two coders [16]. The terms and definitions we use for displays are provided in a glossary (Table 1).

**Table 1:** Glossary of terms

Term	Description	Examples
Visual display	An information visualization [8] such as a chart or table that relates types of information in two-dimensional space	Line chart, table with “traffic light” or red and green indicators, histogram
Content element	Representation of a concept or real entity that is attributed to a visual element of a display	Representation of a hospital, low performance, or a time interval; Internal representation [8]
Content type	A set of content elements that represent the same kind of concept or real entity	Measures, ascribees, performance levels, time intervals
Performance summary	Information about sums, averages, or rates accomplished in a specified time interval	A hospital’s annual readmission rate for 2019, a physician’s average patient satisfaction scores in the last 6 months
Performance summary display (PSD)	A visualization that relates performance levels to other types of information	A bar chart showing a clinic’s monthly patient experience scores over the last 12 months
Chart	A visual display that contains graphical elements, such as points, lines and areas.	Bar chart (also called bar graph), line chart, pie chart
Table	A relational information display that contains cells in rows and columns	This glossary of terms, a spreadsheet
Graph	A chart that contains an x and y axis, along which data are plotted	Bar chart, line chart, box-and-whisker plot, run chart
Measure	A content type representing performance indicators or metrics of care quality and outcomes	Appropriate prescribing of antibiotics, patient-reported blood pressure
Ascribee	A content type representing a feedback recipient or performance comparator	A healthcare professional, hospitals in Michigan, an achievable benchmark
Performance level	A content type representing the result of a quality or outcome measurement process	8%, 13, High, Yes, 24/100, 3.9
Time interval	A content type representing a unit of time	Quarter 3, 2019, October 2006

We developed the proposed method over a period of approximately 3 years, initially in exploratory work during our research team meetings to describe differences between visual displays of feedback, and later in pilot coding to develop and refine a codebook for the method. In team meetings we identified differences between our interpretations that we resolved through discussion. For example, a feedback display that used “traffic light” indicators (Fig. 2) presented challenges for description. One issue concerned whether or not the report contained a single visual display or multiple displays. We resolved this issue by proposing boundary conditions for displays. For example, one condition for identifying an independent display is the identification of a column header or axis label. This decision led us to agree through discussion that the display in Fig. 2 has three separate displays, supported by the fact that the third display shows performance levels only in the form of “Yes” or “No”, in contrast to the percentage values in the other two displays.

A second issue of significant debate in our team was whether or not traffic light indicators represented comparators or performance levels. After repeated failed attempts at achieving agreement through discussion, a team member proposed that we adopt a heuristic of only describing content that had a visual element representing it. This heuristic enabled us to agree that the traffic light indicators did not contain information about a comparator, even though they necessitated the existence of a comparator. Instead, what could be recognized in the visual elements was that the color of the indicators represented categorical levels of performance. Although a comparator of some kind was used to determine the performance level thresholds that determine each indicator’s color, no visual elements of the display could be identified as representing a comparator.

### Step 1: identify displays

We use the term “performance summary display” (PSD) to mean a kind of visualization that relates performance levels to other types of information. In the context of healthcare organizations, PSDs are intended to be communicated to a healthcare professional, team, or organization. Performance summaries often contain multiple tables and charts, some of which may not contain performance information. For example, demographic information about a recipient’s patient population does not indicate a recipient’s performance level, but may be useful for interpreting performance. Therefore, identifying PSDs is a process of firstly recognizing which displays contain performance levels, and secondly determining when multiple independent PSDs are located together and can be further decomposed.

To identify performance levels in a display, we identified terms that indicate the attribution of performance as feedback, such as “Your feedback for January 2019”, a name of a recipient of the feedback report, or another label indicating that the information is the recipient’s own performance (Table 2). To determine whether one or more PSDs is present in a chart or table, one can identify unique titles, axis labels, or column headers that are present (Table 2). These items typically indicate that the feedback recipient is not intended to make comparisons or view trends across regions of a chart, and therefore that multiple PSDs are present. Figures 1 and 2 include examples of two PSDs identified in two example feedback reports.

**Table 2:** Description of content analysis codes

Code	Description
Performance summary display	When to code: 1. The recipient of the performance summary is identifiable 2. The display caption or text of the study indicates that the display was used in a feedback intervention, e.g. “example of a feedback report” 3. Boundary conditions for isolating a display from a set of displays: a. The display has its own title b. The display has its own axis label or column headers When not to code: 1. The display shows only demographic data/ population characteristics that are not about performance 2. The display shows study results about the effect of intervention without indicating that the display was used for feedback 3. There is no other indication that the display is used for performance feedback
Measure set	A set whose elements represent performance metrics or indicators.
Ascribee set	A set whose elements represent things that are ascribed a performance, such as a person, team, organization, or a statistical variable that holds a performance value, such as a peer benchmark, goal, or standard.
Performance set	A set whose elements represent the resulting measurements, decisions, aggregates, and/or calculations related to measured behavior
Time set	A set whose elements represent units of time
Measure element	An element that represents performance metrics or indicators
Ascribee element	An element that represents things that are ascribed a performance, such as a person, team, organization, or a statistical variable that holds a performance value, such as a peer benchmark, goal, or standard.
Performance element	An element that represents the resulting measurements, decisions, aggregates, and/or calculations related to measured behavior
Time element	An element that represents units of time

### Step 2: classify content

Once PSDs have been identified, their content can be classified. Four questions about performance can guide classification of different content types:

1. *What is being measured? Measure* content represents sets of quality metrics or indicators [17] that are sometimes compared in a single display across different clinical practices, or between a process and an outcome for the same clinical practice.
2. *Who is being measured? Ascribee* content represents a feedback recipient and comparator set, such as people, teams, benchmarks and goals, to which performance information is ascribed.
3. *What performance levels are being visualized? Performance level* content represents sets of performance information that can appear as percentages and text, or in colors that may represent a performance category (e.g. red = performance is low). This type of content must be present in a display and must be related to at least one other type of content.
4. *When is performance being measured? Time interval* content represents sets of months, quarters and other time windows in charts that show time-series information.

Figures 1 and 2 provide examples of content types identified in Step 2. Except for performance levels, each content type is optionally included in a visualization. Performance levels are related to at least one other content type, so there must be a minimum of two types of content in a display. For example, in Fig. 1, performance levels are related to ascribees (Your site, Other sites), whereas in Fig. 2, performance levels are related to a set of measures. When one type of information is not visualized, it is typically included in a caption for the chart, or a header in a report. For example, in Fig. 2, which does not visualize time intervals or ascribees, the name of the hospital and the time interval for reported performance appears in a gray box at the top of the report.

### Step 3: identify elements

After classifying PSD content types, content elements in each set can be identified and counted. Content elements may have more than one visual element. For example, in the line chart in Fig. 1, a single performance level element (i.e. a content element) has multiple visual elements. A performance level is represented as a percentage value both by text (e.g. “35.2”) and by the distance between the x-axis and a line. If the text labels for the percentage values were to be removed, the content elements would remain the same because they would still be represented by the distances between the lines and the x-axis of the chart.

### Understanding what was visualized

Having systematically described PSD content for two feedback reports in Figs. 1 and 2, we are now able to recognize differences that may not have been immediately apparent beforehand. For example, we can now recognize that the printed report in Fig. 1 has 4 PSDs, while the clinical quality dashboard in Fig. 2 has 3 PSDs. The displays in Fig. 1 compare ascribees over 4 time intervals per measure, while the displays in Fig. 2 compare performance levels across measures. Figure 1 includes an explicit comparison to “Other sites” while in Fig. 2, no comparator is visualized, instead traffic light indicators show categorical performance levels. The method enables insights about differences between specific displays, but we anticipate that the greater value of the proposed method is in its application to large sets of displays, to understand their implications for effectiveness, theory, and design. We apply the method to a convenience sample of PSDs to illustrate its relevance and applicability to PSDs in studies of A&F on a larger scale.

### Illustration using published a&F studies as exemplars

We illustrate the proposed method in a content analysis [16] of published A&F studies. Published A&F studies describe feedback interventions that commonly use visual displays of performance data in a chart or table. A small proportion of A&F studies include a figure, table, or supplementary file that shows an example performance summary, such as a feedback report or screenshot from a dashboard. Some studies include a complete performance summary, showing how many visual displays were included. Other studies include a partial example of the performance summary, such as showing a single page from a multi-page report, or one screenshot from a dashboard with many tabs.

#### Sample

**Fig. 3:**
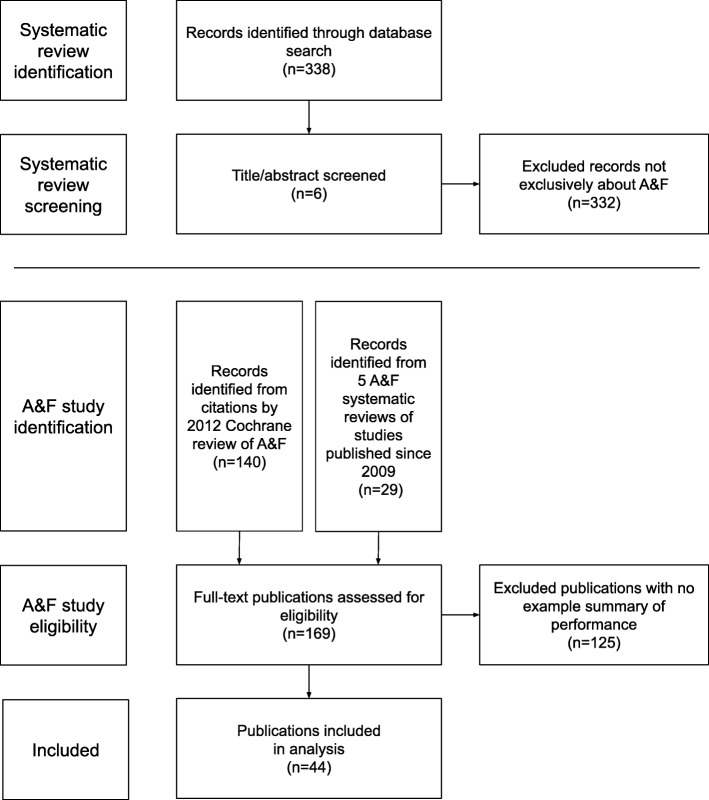
Process for identifying a convenience sample of example performance summaries

We obtained a convenience sample by collecting published A&F studies with example performance summaries (Fig. 3). We identified A&F studies by searching for systematic reviews that were exclusively about A&F. To identify systematic reviews of A&F we screened citations retrieved using the following query in Pubmed: “*Systematic [sb] AND (((“medical audit”[mesh] OR audit [tw]) AND (“feedback”[mesh] OR feedback [tw])) OR “A&F”[tw] OR “e-A&F”[tw])*”. A member of the research team screened citations to identify systematic reviews of A&F. Two members of the research team discussed each systematic review to determine if it included only A&F studies, defined as studies that provided a summary of clinical performance over time to healthcare professionals or teams. This process resulted in a collection of 44 A&F studies for analysis that included an example summary of performance (Fig. 3 and Additional file 1).

#### Step 1: identify displays

The goal of this step was to identify PSDs in example performance summaries, whether partial or complete, from published A&F studies. The unit of analysis was the published A&F study, including the published document and its supplementary material. We developed a single a priori code for a PSD. We developed and revised the code to describe only visual displays that were used in a feedback process. During pilot coding we revised the codebook to describe display boundaries and conditions for decomposing visualizations into multiple PSDs (Table 2).

#### Coding process

Two coders who were graduate students with healthcare professional training (DL and VP) reviewed a draft codebook and completed pilot coding on three A&F studies. We discussed coding differences and iteratively refined the codebook based on pilot coding. Coders received training on the final codes and coded the presence of PSDs in each study. Coders independently coded all studies and displays and then discussed differences with a third team member (ZLL) to reach consensus and to determine the final codes for each example report. Coding for this step was conducted over a period of 1 month.

#### Inter-rater reliability

To assess inter-rater reliability we measured agreement on the number of PSD codes in each study using a two-way mixed, absolute, single-measures intraclass correlation (ICC) statistic. After coding was completed independently for about half of the studies, agreement was in the excellent range [18] at 92.1%.

#### Step 1 analysis

We calculated the frequency of PSDs appearing in each study. Studies without any PSDs were excluded from subsequent analyses. For studies that included at least one PSD, we calculated descriptive statistics for frequency of PSDs in this subsample.

#### Step 2: identify content

Having identified a set of PSDs in Step 1, the goal of Step 2 was to identify the content of each display. The unit of analysis was a single PSD from an example report, drawn from the set of displays identified in Step 1. A PSD included the title and legend relevant to the image, to convey contextual information.

We developed a codebook containing a codes based on the Relational Information Displays framework, interpreted as types of content, in sets for measures, acribees, performance levels, and time intervals (Table 2). We coded each PSD for the presence and absence of each set as a content type. ‘Present’ meant that at least one visual element (e.g. a line, area, color, table row) represented one content element (e.g. a measure, a time interval).

#### Coding process

Coders reviewed the codebook and discussed the codes. During pilot coding, two coders independently coded three PSDs. Coders discussed differences in coding and revised the codebook until agreement on final codes were reached. Coders independently coded the presence of any content type for each PSD. After approximately the first half of the sample was coded, inter-rater reliability was good (*n* = 21, *k* = 0.852). After all displays were coded, the two coders resolved disagreements via discussion with a third team member to determine final codes.

#### Step 2 analysis

We grouped displays by unique combinations of content type. We counted the frequency of displays in each group and for each display format as either a chart or a table. For this analysis, hybrid displays that incorporated graphical elements in tabular form were classified as tables.

#### Step 3: identify elements

The final step was to identify the number of content elements in each content type of each previously coded PSD. The unit of analysis was a content type in a PSD, identified as a measure set, an ascribee set, a performance level set, or a time set (Table 2). We developed a codebook following an identical process described in Step 2, with the exception that codes referred to the members of each set, rather than type of set as a collection of those members. After approximately half of the sample was coded, agreement was very good or excellent for each set (*n* = 102, ICC range: 0.883 to 0.957).

#### Analysis

We calculated descriptive statistics for the sum of elements in each content type in each PSD, and for the overall element totals in each PSD.

## Results

### Step 1: display identification

**Fig. 4:**
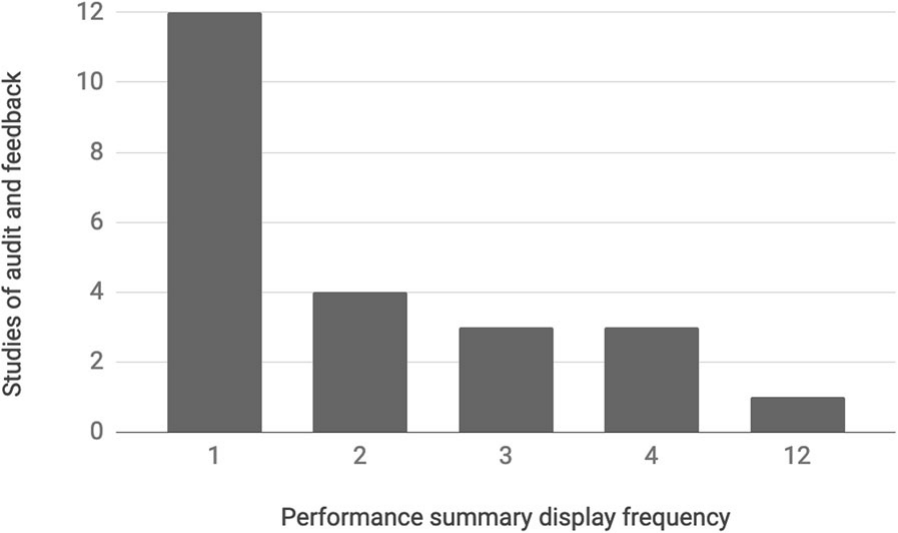
Frequency of performance summary displays identified in example performance summaries

We identified 44 citations of studies for analysis. Of the 44 studies in the sample, 23 (52%) included at least one PSD in an example performance summary. These 23 studies contained an average of 2.2 PSDs (*n* = 53) with most studies’ PSD count ranging from 1 to 4 displays (*n* = 22) and an outlier having 12 PSDs (Fig. 4). The 53 PSDs identified were analyzed in the next step.

### Step 2: content identification

During the coding of 53 PSDs from the previous step, ten were found to have coding issues and were excluded from the subsequent analyses. Four were excluded because they lacked sufficient content to be considered a PSD. Of these four, one display from a clinical quality dashboard [19] was coded as having no performance level set, meaning that by definition it was not a PSD, and the other three were from a single performance summary [20] that was found to have no other content related to performance, therefore they also could not be considered to be PSDs. The remaining six displays were excluded because they were found to have no identifiable content types from the existing codes, due to the fact that their form was a histogram-style display, which relates categories of performance levels (e.g. 0 to 5, 30 to 40) with sums of ascribees (9 hospitals, 2 physicians) that are content types that the codebook was not developed to describe.

In the remaining 43 PSDs, we identified six unique combinations of content types (Table 3). The most frequently occurring content type combinations were ascribee and performance (AP) (*n* = 16), followed by measure, ascribee and performance (MAP) (*n* = 9). The third most common combination was ascribee, performance, and time (APT) (*n* = 7).

**Table 3:** Content types visualized in a feedback intervention study sample (*n* = 43)

Content type	Form	Sub- total	Total	Example Chart	Example Table
Ascribee Performance (AP)	Chart	12	16	Peiris et al. 2015 [21]	Beck et al. 2005 [22]
	Table	4			
Measure Ascribee Performance (MAP)	Chart	2	9	Linder et al. 2010 [19]	Pichert et al. 2013 [23]
	Table	7			
Ascribee Performance Time (APT)	Chart	7	7	Capraro et al. 2012 [24]	None
Measure Performance (MP)	Chart	1	8	Peiris et al. 2015 [21]	Gude et al. 2016 [25]
	Table	7			
Performance Time (PT)	Chart	2	2	Blomberg et al. 2016 [26]	None
Measure Ascribee Performance Time (MAPT)	Table	1	1	None	Rantz et al. 2001 [27]
Total of studies			43		

Content types are combinations of Measure, Ascribee, Performance level or Time interval (MAPT) sets

### Step 3: element counts

Coding the content elements revealed that one PSD had no performance level visual elements, and therefore no content elements, because it showed a blank template rather than an example performance summary with data in it. We excluded this single PSD and analyzed the remaining 42 PSDs (Table 4). Content element totals for any content type ranged from 4 to 138. The average number of elements in a content type was approximately 6 for measure, ascribee, and time intervals, while the average for performance levels was 13.8 (Table 4).

**Table 4:** Frequency of visualized content and element counts in a sample of performance summary displays (*n* = 42)

	Measure	Ascribee	Performance	Time
Content type frequency (n) (%)	17 (40.4%)	32 (76.2%)	42 (100%)	10 (23.8%)
Element count mean	6.5	6.8	13.8	6.2
Element count min	1	2	1	5
Element count max	20	46	92	10

## Discussion

This study proposes a method for the systematic description of visualizations of clinical performance. We applied this method in the context of A&F studies, from a sample of published example performance summaries. A series of directed content analyses demonstrated the systematic description of PSDs in three steps: 1) identify displays, 2) classify content, and 3) identify elements. Two coders completed each step with good to excellent agreement. Using the method, we systematically identified 6 unique combinations of types of visualized content, and we were able to describe their prevalence within a limited convenience sample (Table 3). The inclusion of measures, ascribees, and time intervals varied across the 6 content types.

These findings are significant because they demonstrate the ability of the method to support a systematic analysis of visualizations that has many potential applications. From an empirical perspective, the method enables the content of displays to be controlled for experimentation and evidence synthesis to learn about relationships between PSDs and feedback effectiveness. From a theoretical perspective, display content can be analyzed to understand implications for known mechanisms of influence. For example, Feedback Intervention Theory [4] could be used to contrast the effects of displays having vs lacking ascribee content as a form of normative comparison. From a design perspective, the testing of prototype PSDs in feedback reports and dashboards could be intentionally varied across content types to ensure that a broader range of information is considered for meeting users’ information needs and to maximize PSD usability.

A secondary review of A&F studies has described design and reporting elements of A&F interventions, identifying the use of graphical elements as an important characteristic [5]. We build on this work by using a visualization framework to inform the identification of graphical elements, and relate these characteristics to both tables and charts. We further develop the ability to specify the content of visual displays, where previous studies investigating graphical literacy and numeracy of clinical practice data have investigated visual displays that are specified at the level of the whole display [13, 15]. For example, bar graphs and pictographs were identified as optimal in a study of anesthesiologists, but these “whole display” level descriptions do not speak to hybrid display types, such as tables that use graphical elements in a traffic light or “red, amber, green”-style display [28].

We have demonstrated that a method based on the Relational Information Displays framework can be used to systematically describe the use of visualizations in feedback reports and quality dashboards, and to describe basic components of these visualizations. Our method also may be useful for the management of performance visualizations in healthcare organizations. For example, the method could be used to review the display types and content deployed in a dashboard across an organization, to better understand characteristics of preferred displays at a lower-level than the whole display.

There were several limitations for our analysis. We used a small convenience sample that is not generalizable for PSD use in A&F. The small sample contains example performance summaries that are not necessarily complete, with many containing only one page or screenshot from a larger set of pages in a report or dashboard. Furthermore, the sample is from research studies that span 4 decades, including PSDs that were generated using obsolete software and technology. The findings therefore are intended only to be interpreted as a demonstration of the description that is possible, not as a characterization of PSD use in A&F.

Another limitation is that, although we identified 6 unique combinations of content types, the codebook did not support the coding of PSD content in histograms. We expect that an important next step for this work will be to develop systematic description of the content of histograms to enable the inclusion of these types of displays in future analyses of PSDs.

A further limitation for our analysis is in coding issues that led to the exclusion of displays that were mis-identified as PSDs, based on our codes, once we moved from one step to the next. These errors reflect the complexity of interpreting displays, which is problematic in itself, given that coding was done by trained healthcare professionals, but also reveal the ability of the method to provide insight that is difficult to gain without a systematic approach to describing visualized content.

Finally, the proposed method is not evidence-based and was refined during the pilot coding phases of each step. Nevertheless, we anticipate that the proposed method demonstrates a significant advance for A&F researchers who plan to evaluate feedback interventions that use visual displays. We anticipate that this method can provide a foundation for the systematic study of an important component of feedback interventions, which is its visualized information content.

## Conclusion

Visualization frameworks can be used to understand the use of visual displays, as well as to systematically describe their content. The proposed method appears to be feasible to use as a systematic approach to describing visual displays of clinical performance. The key implications of the method are that it offers more granular and consistent description for empirical, theoretical, and design studies about the information content of feedback interventions.

## Supplementary information

**Additional file 1.** Systematic reviews, A&F studies, and number of PSDs in sample. Table containing counts of PSDs and references for their associated A&F studies and systematic reviews.

## Data Availability

Data and materials are publicly available at: 10.6084/m9.figshare.11932860.

## Abbreviations

A&F: Audit and Feedback
PSD: Performance Summary Display
