# Supplementary material for: What was visualized? A method for describing content of performance summary displays in feedback interventions
Source: BMC Med Res Methodol. 2020 Apr 23;20:90. doi: 10.1186/s12874-020-00951-x (PMC7181510; doi:10.1186/s12874-020-00951-x)
Supplement: Supplementary file 1 — Additional file 1. Systematic reviews, A&F studies, and number of PSDs in sample. Table containing counts of PSDs and references for their associated A&F studies and systematic reviews. [file 12874_2020_951_MOESM1_ESM.pdf]

Additional file 1: Systematic reviews, A&F studies, and number of PSDs in sample

Systematic review	A&F Study	Number of PSDs
Sykes et al., 2018 [1]	Wilson et al., 2015 [2]	0
	Rooney 2014 [3]	0
	Mills et al., 2014 [4]	0
	Dawson 2014 [5]	0
	Dodd 2013 [6]	0
Le Grand Rogers et al,. 2015 [7]	Nguyen et al., 2014 [8]	0
	Pichert et al., 2013 [9]	4
	Wu et al., 2013 [10]	0
	Volpe et al., 2012 [11]	0
	Capraro et al., 2012 [12]	4
	Welch 2011 [13]	0
	McIntosh et al., 2011 [14]	0
Boatin et al,. 2017 [15]	Maneschi et al., 2015 [16]	0
	Blomberg 2016 [17]	1
	Reis et al., 2015 [18]	0
	Svelato et al., 2014 [19]	0
	Scarella et al., 2011 [20]	0
Tuti et al., 2017 [21]	Gude et al., 2016 [22]	3
	Peiris et al., 2015 [23]	2
	Guldberg et al., 2011 [24]	0
	Carney et al., 2011 [25]	0
	Linder et al., 2010 [26]	4
Ivers et al 2012 [27]	Beck et al., 2005 [28]	12
	Bentz et al., 2007 [29]	4
	Kritchevsky et al., 2008 [30]	1
	Ornstein et al., 2004 [31]	1
	Verstappen 2004 [32]	0

	Wadland et al., 2007 [33]	2
	Pimlott et al., 2003 [34]	1
	Rantz et al.,2001 [35]	2
	Søndergaard et al., 2002 [36]	1
	Lobach 1996 [37]	1
	Winickoff et al., 1984 [38]	1
	Balas et al., 1998 [39]	1
	Gehlbach et al., 1984 [40]	1
	Hershey et al.,1988 [41]	1
	Hershey et al., 1986 [42]	2
	Tierney et al., 1986 [43]	3
Tinmouth et al 2016 [44]	Kaminski et al., 2016 [45]	0
	Keswani et al., 2015 [46]	1
	Fraser et al., 2013 [47]	0
	Kahi et al., 2013 [48]	0
	Coe et al., 2013 [49]	0
	Lin et al., 2010 [50]	0
Total	44	53

References

1. Sykes MJ, McAnuff J, Kolehmainen N. When is audit and feedback effective in dementia care? A systematic review. Int J Nurs Stud. 2018 Mar;79:27–35.
2. Wilson LS, Devitt P, Hally O. Standards of prescription writing in a long-term psychogeriatric unit: a series of clinical audits. Ir J Psychol Med. 2015 Jun;32(2):197–204.
3. Rooney CR. Management of physical aggression in the dementia resident in an 18 bed secure dementia unit: A best practice implementation project: JBI Database Syst Rev Implement Rep. 2014 Aug;12(8):394–410.
4. Mills JKA, Minhas JS, Robotham SL. An assessment of the dementia CQUIN – an audit of improving compliance. Dementia. 2014 Sep;13(5):697–703.
5. Dawson E. Reducing medicines administration errors on dementia units: Audit. Prog Neurol Psychiatry. 2014 Jan;18(1):20–3.
6. Dodd C. An audit investigating cognitive assessment of emergency general surgical admissions in elderly patients in Sheffield, UK. Int J Surg. 2013 Oct;11(8):649.
7. Rogers RLG, Narvaez Y, Venkatesh AK, Fleischman W, Hall MK, Taylor RA, Hersey D, Sette L, Melnick ER. Improving emergency physician performance using audit and feedback: a systematic review. Am J Emerg Med. 2015 Oct 1;33(10):1505–14.
8. Nguyen MC, Richardson DM, Hardy SG, Cookson RM, Mackenzie RS, Greenberg MR, Glenn-Porter B,

Kane BG. Computer-based reminder system effectively impacts physician documentation. Am J Emerg Med. 2014 Jan 1;32(1):104–6.
9. Pichert JW, Moore IN, Karrass J, Jay JS, Westlake MW, Catron TF, Hickson GB. An intervention model that promotes accountability: peer messengers and patient/family complaints. Jt Comm J Qual Patient Saf. 2013 Oct;39(10):435–46.
10. Wu K-H, Cheng F-J, Li C-J, Cheng H-H, Lee W-H, Lee C-W. Evaluation of the effectiveness of peer pressure to change disposition decisions and patient throughput by emergency physician. Am J Emerg Med. 2013 Mar;31(3):535–9.
11. Volpe D, Harrison S, Damian F, Rachh P, Kahlon PS, Morrissey L, Mack J, Akenroye A, Stack AM. Improving timeliness of antibiotic delivery for patients with fever and suspected neutropenia in a pediatric emergency department. Pediatrics. 2012 Jul;130(1):e201-210.
12. Capraro A, Stack A, Harper MB, Kimia A. Detecting unapproved abbreviations in the electronic medical record. Jt Comm J Qual Patient Saf. 2012 Apr;38(4):178–83.
13. Welch S, Dalto J. Improving Door-to-Physician Times in 2 Community Hospital Emergency Departments. Am J Med Qual. 2011 Mar 1;26(2):138–44.
14. McIntosh KA, Maxwell DJ, Pulver LK, Horn F, Robertson MB, Kaye KI, Peterson GM, Dollman WB, Wai A, Tett SE. A quality improvement initiative to improve adherence to national guidelines for empiric management of community-acquired pneumonia in emergency departments. Int J Qual Health Care J Int Soc Qual Health Care. 2011 Apr;23(2):142–50.
15. Boatin AA, Cullinane F, Torloni MR, Betrán AP. Audit and feedback using the Robson classification to reduce caesarean section rates: a systematic review. BJOG Int J Obstet Gynaecol. 2017 Jun 11;125(1):36–42.
16. Maneschi F, Algieri M, Perrone S, Nale R, Sarno M. Cesarean 10-group classification: a tool for delivery ward clinical management. Minerva Ginecol. 2015 Jan 14;67.
17. Blomberg M. Avoiding the first cesarean section--results of structured organizational and cultural changes. Acta Obstet Gynecol Scand. 2016 May;95(5):580–6.
18. Reis Z, Aguiar R, Rodrigues dos Santos Junior M, Rodrigues dos Santos M, Gaspar J. IMPLEMENTATION OF THE CESAREAN BIRTHS REVIEW USING THE TEN GROUP ROBSON'S CLASSIFICATION AND ITS IMMEDIATE EFFECTS ON THE RATE OF CAESAREANS, AT AN UNIVERSITY HOSPITAL. In 2015.
19. Svelato A, Meroni M, Poli M, Perino A, Spinoso R, Ragusa A. How to reduce caesarean sections in first four Robson's classess. In: BJOG An International Journal of Obstetrics & Gynaecology. 2014.
20. Scarella A, Chamy V, Sepúlveda M, Belizán JM. Medical audit using the Ten Group Classification System and its impact on the cesarean section rate. Eur J Obstet Gynecol Reprod Biol. 2011 Feb;154(2):136–40.
21. Tuti T, Nzinga J, Njoroge M, Brown B, Peek N, English M, Paton C, van der Veer SN. A systematic review of electronic audit and feedback: intervention effectiveness and use of behaviour change theory. Implement Sci. 2017 May 12;12:61.
22. Gude WT, van Engen-Verheul MM, van der Veer SN, Kemps HMC, Jaspers MWM, de Keizer NF, Peek N. Effect of a web-based audit and feedback intervention with outreach visits on the clinical performance of multidisciplinary teams: a cluster-randomized trial in cardiac rehabilitation. Implement Sci IS [Internet]. 2016 Dec 9 [cited 2018 May 23];11. Available from: https://www.ncbi.nlm.nih.gov/pmc/articles/PMC5148845/
23. Peiris D, Usherwood T, Panaretto K, Harris M, Hunt J, Patel B, Zwar N, Redfern J, MacMahon S, Colagiuri S, Hayman N, Patel A. The Treatment of cardiovascular Risk in Primary care using Electronic Decision suppOrt (TORPEDO) study: intervention development and protocol for a cluster randomised, controlled trial of an electronic decision support and quality improvement intervention in Australian primary healthcare. BMJ Open [Internet]. 2012 Nov 22 [cited 2018 May 8];2(6). Available from: https://www.ncbi.nlm.nih.gov/pmc/articles/PMC3533097/
24. Guldberg TL, Vedsted P, Kristensen JK, Lauritzen T. Improved quality of Type 2 diabetes care following electronic feedback of treatment status to general practitioners: a cluster randomized controlled trial. Diabet Med. 2011 Mar 1;28(3):325–32.
25. Carney PA, Bowles EJA, Sickles EA, Geller BM, Feig SA, Jackson S, Brown D, Cook A, Yankaskas BC, Miglioretti DL, Elmore JG. Using a tailored web-based intervention to set goals to reduce unnecessary

recall. Acad Radiol. 2011 Apr;18(4):495–503.
26. Linder JA, Schnipper JL, Tsurikova R, Yu DT, Volk LA, Melnikas AJ, Palchuk MB, Olsha-Yehiav M, Middleton B. Electronic health record feedback to improve antibiotic prescribing for acute respiratory infections. Am J Manag Care. 2010 Dec;16(12 Suppl HIT):e311-319.
27. Ivers N, Jamtvedt G, Flottorp S, Young JM, Odgaard-Jensen J, French SD, O'Brien MA, Johansen M, Grimshaw J, Oxman AD. Audit and feedback: effects on professional practice and healthcare outcomes. Cochrane Database Syst Rev. 2012;6:CD000259.
28. Beck CA, Richard H, Tu JV, Pilote L. Administrative Data Feedback for Effective Cardiac Treatment: AFFECT, a cluster randomized trial. JAMA. 2005 Jul 20;294(3):309–17.
29. Bentz CJ, Bayley KB, Bonin KE, Fleming L, Hollis JF, Hunt JS, LeBlanc B, McAfee T, Payne N, Siemienczuk J. Provider feedback to improve 5A's tobacco cessation in primary care: a cluster randomized clinical trial. Nicotine Tob Res Off J Soc Res Nicotine Tob. 2007 Mar;9(3):341–9.
30. Kritchevsky SB, Braun BI, Bush AJ, Bozikis MR, Kusek L, Burke JP, Wong ES, Jernigan J, Davis CC, Simmons B, TRAPE Study Group. The effect of a quality improvement collaborative to improve antimicrobial prophylaxis in surgical patients: a randomized trial. Ann Intern Med. 2008 Oct 7;149(7):472–80, W89-93.
31. Ornstein S, Jenkins RG, Nietert PJ, Feifer C, Roylance LF, Nemeth L, Corley S, Dickerson L, Bradford WD, Litvin C. A multimethod quality improvement intervention to improve preventive cardiovascular care: a cluster randomized trial. Ann Intern Med. 2004 Oct 5;141(7):523–32.
32. Verstappen WHJM, van der Weijden T, Dubois WI, Smeele I, Hermsen J, Tan FES, Grol RPTM. Improving test ordering in primary care: the added value of a small-group quality improvement strategy compared with classic feedback only. Ann Fam Med. 2004 Dec;2(6):569–75.
33. Wadland WC, Holtrop JS, Weismantel D, Pathak PK, Fadel H, Powell J. Practice-based referrals to a tobacco cessation quit line: assessing the impact of comparative feedback vs general reminders. Ann Fam Med. 2007 Apr;5(2):135–42.
34. Pimlott NJG, Hux JE, Wilson LM, Kahan M, Li C, Rosser WW. Educating physicians to reduce benzodiazepine use by elderly patients: a randomized controlled trial. CMAJ Can Med Assoc J J Assoc Medicale Can. 2003 Apr 1;168(7):835–9.
35. Rantz MJ, Popejoy L, Petroski GF, Madsen RW, Mehr DR, Zwygart-Stauffacher M, Hicks LL, Grando V, Wipke-Tevis DD, Bostick J, Porter R, Conn VS, Maas M. Randomized clinical trial of a quality improvement intervention in nursing homes. The Gerontologist. 2001 Aug;41(4):525–38.
36. Søndergaard J, Andersen M, Vach K, Kragstrup J, Maclure M, Gram LF. Detailed postal feedback about prescribing to asthma patients combined with a guideline statement showed no impact: a randomised controlled trial. Eur J Clin Pharmacol. 2002 May;58(2):127–32.
37. Lobach DF. Electronically distributed, computer-generated, individualized feedback enhances the use of a computerized practice guideline. Proc Conf Am Med Inform Assoc AMIA Annu Fall Symp AMIA Fall Symp. 1996;493–7.
38. Winickoff RN, Coltin KL, Morgan MM, Buxbaum RC, Barnett GO. Improving physician performance through peer comparison feedback. Med Care. 1984 Jun;22(6):527–34.
39. Balas EA, Boren SA, Hicks LL, Chonko AM, Stephenson K. Effect of linking practice data to published evidence. A randomized controlled trial of clinical direct reports. Med Care. 1998 Jan;36(1):79–87.
40. Gehlbach SH, Wilkinson WE, Hammond WE, Clapp NE, Finn AL, Taylor WJ, Rodell MS. Improving drug prescribing in a primary care practice. Med Care. 1984 Mar;22(3):193–201.
41. Hershey CO, Goldberg HI, Cohen DI. The effect of computerized feedback coupled with a newsletter upon outpatient prescribing charges. A randomized controlled trial. Med Care. 1988 Jan;26(1):88–94.
42. Hershey CO, Porter DK, Breslau D, Cohen DI. Influence of simple computerized feedback on prescription charges in an ambulatory clinic. A randomized clinical trial. Med Care. 1986 Jun;24(6):472–81.
43. Tierney WM, Hui SL, McDonald CJ. Delayed feedback of physician performance versus immediate reminders to perform preventive care. Effects on physician compliance. Med Care. 1986 Aug;24(8):659–66.
44. Tinmouth J, Patel J, Hilsden RJ, Ivers N, Llovet D. Audit and feedback interventions to improve endoscopist performance: Principles and effectiveness. Best Pract Res Clin Gastroenterol. 2016 Jun 1;30(3):473–85.

45. Kaminski MF, Anderson J, Valori R, Kraszewska E, Rupinski M, Pachlewski J, Wronska E, Bretthauer M, Thomas-Gibson S, Kuipers EJ, Regula J. Leadership training to improve adenoma detection rate in screening colonoscopy: a randomised trial. Gut. 2016 Apr 1;65(4):616–24.
46. Keswani RN, Yadlapati R, Gleason KM, Ciolino JD, Manka M, O'Leary KJ, Barnard C, Pandolfino JE. Physician Report Cards and Implementing Standards of Practice Are Both Significantly Associated With Improved Screening Colonoscopy Quality. Am J Gastroenterol. 2015 Aug;110(8):1134–9.
47. Fraser AG, Gamble GD, Rose TR, Dunn JP. Colonoscopy audit over 10 years—what can be learnt? 2013;126(1382):11.
48. Kahi CJ, Ballard D, Shah AS, Mears R, Johnson CS. Impact of a quarterly report card on colonoscopy quality measures. Gastrointest Endosc. 2013 Jun 1;77(6):925–31.
49. Coe SG, Crook JE, Diehl NN, Wallace MB. An Endoscopic Quality Improvement Program Improves Detection of Colorectal Adenomas. Am J Gastroenterol. 2013 Feb;108(2):219–26.
50. Lin OS, Kozarek RA, Arai A, Gluck M, Jiranek GC, Kowdley KV, McCormick SE, Schembre DB, Soon M-S, Dominitz JA. The effect of periodic monitoring and feedback on screening colonoscopy withdrawal times, polyp detection rates, and patient satisfaction scores. Gastrointest Endosc. 2010 Jun 1;71(7):1253–9.
